# Differential Effects of Hazardous Drinking on Post-Traumatic Stress Disorder Outcomes Across Two Prolonged Exposure Treatment Formats †

**DOI:** 10.3390/bs15070954

**Published:** 2025-07-15

**Authors:** Casey L. Straud, Kiara H. Buccellato, Edna B. Foa, Lily A. Brown, Carmen P. McLean, Tabatha H. Blount, Richard P. Schobitz, Bryann B. DeBeer, Joseph Mignogna, Brooke A. Fina, Brittany N. Hall-Clark, Christian C. Schrader, Jeffrey S. Yarvis, Vanessa M. Jacoby, Wyatt R. Evans, Brett T. Litz, Eric C. Meyer, Barbara L. Niles, Stacey Young-McCaughan, Terence M. Keane, Alan L. Peterson

**Affiliations:** 1 Department of Psychiatry and Behavioral Sciences, University of Texas Health Science Center at San Antonio, San Antonio, TX 78229, USA; buccellato@uthscsa.edu (K.H.B.); blountt@uthscsa.edu (T.H.B.); fina@uthscsa.edu (B.A.F.); hallclark@uthscsa.edu (B.N.H.-C.); jacobyv@uthscsa.edu (V.M.J.); wyattrevans@gmail.com (W.R.E.); youngs1@uthscsa.edu (S.Y.-M.); petersona3@uthscsa.edu (A.L.P.); 2 Department of Psychology, University of Texas at San Antonio, San Antonio, TX 78249, USA; 3 Research and Development Service, South Texas Veterans Health Care System, San Antonio, TX 78229, USA; 4 Center for the Treatment and Study of Anxiety, Department of Psychiatry, University of Pennsylvania, Philadelphia, PA 19104, USA; foa@pennmedicine.upenn.edu (E.B.F.); lilybr@pennmedicine.upenn.edu (L.A.B.); 5 Dissemination and Training Division, National Center for PTSD, VA Palo Alto Health Care System, Menlo Park, CA 94025, USA; carmen.mclean4@va.gov; 6 Department of Psychiatry and Behavioral Sciences, Stanford University, Stanford, CA 94305, USA; 7 Department of Behavioral Health, Brooke Army Medical Center, Joint Base San Antonio-Fort Sam Houston, TX 78234, USA; richard.schobitz@hhs.gov; 8 U.S. Public Health Service Commissioned Corps Headquarters, Rockville, MD 20852, USA; 9 Rocky Mountain MIRECC for Suicide Prevention, U.S. Department of Veterans Affairs, Aurora, CO 80045, USA; bryann.debeer@va.gov (B.B.D.); joseph.mignogna@va.gov (J.M.); 10 Department of Physical Medicine and Rehabilitation, University of Colorado Anschutz Medical Campus, Aurora, CO 80045, USA; 11 Department of Behavioral Health, Carl R. Darnall Army Medical Center, Fort Hood, TX 76544, USA; christian@originstory.health (C.C.S.); jyarvis@tulane.edu (J.S.Y.); 12 School of Social Work, Tulane University, New Orleans, LA 70112, USA; 13 VA North Texas Health Care System, Dallas, TX 75216, USA; 14 Massachusetts Veterans Epidemiology Research and Information Center, VA Boston Healthcare System, Boston, MA 02130, USA; litzb@bu.edu; 15 Department of Psychiatry, Boston University Chobanian and Avedisian School of Medicine, Boston, MA 02118, USA; barbara.niles@va.gov (B.L.N.); terry.keane@va.gov (T.M.K.); 16 Department of Counseling and Behavioral Health, University of Pittsburgh, Pittsburgh, PA 15260, USA; ecm77@pitt.edu; 17 Behavioral Science Division, National Center for PTSD, VA Boston Healthcare System, Boston, MA 02130, USA

**Keywords:** post-traumatic stress disorder, hazardous drinking, prolonged exposure, intensive outpatient program treatment format, massed treatment format

## Abstract

Individuals with post-traumatic stress disorder (PTSD) are at increased risk for hazardous drinking, which often complicates treatment and affects trauma-focused psychotherapy outcomes. The present study is an exploratory, secondary analysis investigating differential effects of prolonged exposure (PE) among those with and without hazardous drinking and whether treatment outcomes varied across these groups as a function of PE format. Data used were from a randomized controlled trial that examined two daily, compressed formats of PE treatment for PTSD (massed and intensive outpatient program) in military personnel and veterans (N = 234). Individuals without hazardous drinking had greater PTSD symptom reductions compared to those with hazardous drinking (*d* = 0.42, *p* = 0.001). However, the hazardous drinking group also demonstrated significant reductions in PTSD (*d* = 1.46, *p* < 0.001) following treatment, as well as in the number of drinks per week (*d* = 0.63, *p* = 0.025) at the 6-month follow-up. There was no significant difference in treatment engagement based on drinking classification and outcomes did not vary based on PE format. The findings suggest that PE is an appropriate treatment for individuals with PTSD and hazardous drinking. However, group differences in PTSD symptom reductions indicate concurrent hazardous drinking reduces treatment benefits of PE.

## 1. Introduction

Post-traumatic stress disorder (PTSD) is a highly prevalent mental health condition that affects between 4 and 17% of U.S. military service members and veterans who have deployed in support of post-9/11 operations (Krull et al., 2022; Richardson et al., 2010). PTSD is characterized by a constellation of symptoms that emerge following exposure to a potentially traumatic event, which can include directly experiencing or witnessing actual or threatened death, serious injury, or sexual violence, or learning about such events occurring to a significant other (American Psychiatric Association [APA], 2013). The core symptoms of PTSD are re-experiencing, avoidance, negative alterations in cognition and mood, and alterations in arousal and reactivity. Overall, the impact of PTSD can lead to significant long-term functional impairments, including difficulties in social, occupational, and emotional functioning (Hoge et al., 2007).

Fortunately, evidence-based treatments for PTSD exist and are well-established. Prolonged Exposure (PE) is an evidence-based, trauma-focused cognitive-behavioral therapy for PTSD that includes two primary exposure components to process the trauma memory: imaginal exposure (repeatedly recounting and processing the traumatic memory in a safe environment) and in vivo exposure (systematically approaching real-world situations considered safe but that have been avoided due to fear or trauma reminders; Foa et al., 2019). The theoretical rationale for PE is based on Emotional Processing Theory (Foa & Kozak, 1985), which suggests PTSD is maintained through negative reinforcement, where avoidance impacts the ability to unlearn trauma associations (i.e., inhibitory learning). Exposure interrupts avoidance patterns that maintain PTSD symptoms and promotes processing the trauma and PTSD symptom reductions. Standard PE typically includes 10–15 weekly sessions (60–90 min) delivered over several months. Research has shown that PE can be delivered in a compressed, daily (massed) format that is similarly effective as standard PE, but with significantly less treatment dropout (Foa et al., 2018).

Hazardous drinking is a pattern of alcohol consumption that exceeds conventional guidelines of low-risk drinking and increases risk of physical, mental, or social problems (Reid et al., 1999). Hazardous drinking is one of the most prevalent co-occurring problems associated with PTSD, with approximately 35–50% of individuals with PTSD also meeting criteria for hazardous drinking or alcohol use disorder (Debell et al., 2014; Pietrzak et al., 2011). Post-9/11 U.S. veterans with PTSD are twice as likely to report hazardous drinking compared to veterans without PTSD (Jakupcak et al., 2010). The self-medication hypothesis offers a framework to explain the association between PTSD and hazardous drinking, where the function of alcohol use is to mitigate symptoms of PTSD and associated distress (Khantzian, 1997). This maladaptive coping strategy may provide short-term relief, but long-term, chronic drinking can reinforce a reciprocal pattern that leads to a more severe, complex symptom presentation, impaired functioning, poorer health, and lower quality of life (Blanco et al., 2013; Coffey et al., 2016; Norman et al., 2018). The co-occurrence of PTSD and substance use disorder (to include alcohol use disorder) is associated with greater PTSD and substance use symptom burden, lower treatment engagement, and reduced treatment benefits compared to individuals with either disorder alone (Coffey et al., 2016; McCauley et al., 2012; Roberts et al., 2015). Collectively, these findings highlight the unique challenges to effective treatment engagement and response within this population.

Clinicians commonly report concerns that co-occurring PTSD and hazardous drinking is more difficult to treat; that evidence-based, trauma-focused psychotherapy for PTSD may not be effective; and that trauma-focused interventions may even increase PTSD and drinking symptoms (Becker et al., 2004; Blanco et al., 2013; Norman et al., 2018; Schäfer & Najavits, 2007). Some clinicians are reluctant to engage in trauma-focused psychotherapy because of concern that trauma processing may exacerbate PTSD symptoms, increase substance use or cravings, and ultimately lead to increased risk of treatment dropout or limited treatment effectiveness (Back et al., 2009; Becker et al., 2004; Najavits, 2002; Riggs et al., 2003). These concerns have historically contributed to the exclusion of individuals with active substance misuse from PTSD interventions and PTSD treatment research with the recommendation that the individual address the substance misuse prior to engaging in a PTSD intervention. However, a growing body of evidence has shown that individuals receiving evidence-based, trauma-focused psychotherapy, whether delivered alone or integrated with a substance use intervention, can achieve clinically meaningful reductions in PTSD and substance use (Hien et al., 2023; Roberts et al., 2015). Further, research has found that many individuals with PTSD and substance misuse prefer to immediately address both problems (integrated) as opposed to a sequential approach that delays PTSD treatment (Back et al., 2006). U.S. Veterans Affairs (VA) and Department of Defense (DoD) clinical practice guidelines also stipulate the importance of access to evidence-based care for PTSD, regardless of co-occurring substance use (VA/DoD, 2023).

Integrated treatment approaches, such as concurrent treatment of PTSD and substance use disorders using prolonged exposure (COPE), have emerged as a gold standard for addressing co-occurring PTSD and substance use disorder (Back et al., 2014). COPE combines evidence-based, cognitive-behavioral interventions for substance misuse with PE for PTSD, offering a framework for concurrently targeting both conditions. COPE is associated with reductions in PTSD and substance use severity in civilian and military samples (Back et al., 2019; Persson et al., 2017). Additionally, Foa et al. (2013) conducted a randomized clinical trial examining the efficacy of PE, naltrexone, and their combination for comorbid PTSD and alcohol use. In that study, all treatment groups showed significant improvement in PTSD, while those who received naltrexone also demonstrated improvements in alcohol outcomes. Collectively, these findings suggest that combined interventions for PTSD and substance use are safe and effective and that PE is not associated with symptom exacerbation among individuals with both conditions.

Although prior research has demonstrated the benefits of PE for PTSD and alcohol use when delivered as an integrated treatment, there has been limited research focused on the effects of PE alone for individuals with co-occurring PTSD and hazardous drinking, particularly when delivered in a massed treatment format of daily sessions over several weeks. However, research on cognitive processing therapy (CPT)—another trauma-focused psychotherapy for PTSD—has shown that individuals with hazardous drinking can benefit to the same degree as those without hazardous drinking when the intervention is delivered alone, with comparable PTSD reductions and treatment engagement (Dondanville et al., 2019; LoSavio et al., 2023; Straud et al., 2021). These studies have also found that individuals with hazardous drinking have significant reductions in drinking following treatment, despite the fact that CPT does not explicitly address drinking reductions as a treatment goal. Likewise, Held et al. (2021) demonstrated that individuals with hazardous drinking who engaged in a compressed, daily massed treatment format of CPT also had PTSD improvements and drinking reductions, with similar treatment engagement rates compared to participants without hazardous drinking. Overall, these findings are promising regarding the benefits of trauma-focused psychotherapies among individuals with co-occurring PTSD and hazardous drinking. However, it is important to determine if CPT results generalize to PE.

The present study is an exploratory, secondary analysis of a parent randomized controlled trial that investigated the effects of two daily, compressed formats of PE treatment to reduce PTSD symptoms: (1) massed (M-PE) and (2) intensive outpatient program (IOP-PE; Peterson et al., 2018, 2023). M-PE was a compressed version of standard PE therapy delivered in 15 daily sessions over 3 weeks. The IOP-PE was a full-day treatment program that included 15 daily PE sessions over 3 weeks and eight additional components hypothesized to enhance and sustain treatment outcomes (described in the Section 2). Study aims were to examine PTSD treatment outcome differences in a sample of individuals with hazardous drinking compared to those without hazardous drinking. Secondary aims evaluated drinking reductions and treatment engagement (attrition). We hypothesized that military personnel with PTSD and baseline hazardous drinking would demonstrate less PTSD improvement compared to individuals without hazardous drinking (Hypothesis 1a). We also hypothesized that participants with hazardous drinking randomized to IOP-PE would demonstrate greater improvements compared to those with hazardous drinking in M-PE due to the additional components of IOP-PE, which may provide a higher level of support to address co-occurring PTSD and hazardous drinking (Hypothesis 1b). Second, we hypothesized that individuals with hazardous drinking would demonstrate significant drinking reductions following PE regardless of treatment format, but the IOP-PE would have greater improvements compared to M-PE (Hypothesis 2). Finally, we hypothesized that individuals with hazardous drinking would be more likely to drop out of treatment compared to those without hazardous drinking (Hypothesis 3a). However, we hypothesized that participants with hazardous drinking randomized to IOP-PE would be more likely to complete treatment compared to those with hazardous drinking randomized to M-PE (Hypothesis 3b).

## 2. Materials and Methods

### 2.1. Participants and Procedures

This study is a secondary analysis that used data collected from a randomized controlled trial comparing the efficacy of M-PE and IOP-PE delivered over 3 weeks for PTSD (Peterson et al., 2018, 2023). The sample was comprised of active-duty military service members and veterans who deployed in support of post-9/11 U.S. military combat operations. Individuals were, on average, 39 years old (SD = 7.72), predominantly men (77.8%), married (64.5%), and representative of diverse racial backgrounds (Table 1). All participants experienced at least one deployment-related Criterion A event and met diagnostic criteria for PTSD on the Clinician Administer PTSD Scale (CAPS-5) for the Diagnostic and Statistical Manual of Mental Disorders, 5th Edition (DSM-5; Weathers et al., 2013a).

Individuals were recruited from four sites in Texas (two U.S. military treatment facilities and two U.S. Department of Veterans Affairs healthcare facilities) and randomly assigned to IOP-PE (*n* = 117) or M-PE (*n* = 117). Participants completed assessments at baseline and at 1-, 3-, and 6-months post-treatment follow-up, with select self-report measures administered weekly during treatment. Baseline and follow-up assessments were administered by master’s- or doctoral-level independent evaluators who were blinded to the treatment arm. The parent study was conducted in accordance with the Declaration of Helsinki and approved by the Institutional Review Boards (IRB; #16-422H, first approved 22 September 2016). The University of Texas Health Science Center at San Antonio (San Antonio, TX, USA) served as the IRB-of-record and maintained the core protocol. The U.S. Army Medical Research and Development Command Office of Human Research Protection reviewed the regulatory determinations for this study.

Study therapists had master’s or doctoral degrees in social work or psychology and received extensive training in PE prior to delivering the intervention. PE was the foundation for both treatment protocols. PE is an evidence-based, cognitive-behavioral psychotherapy for PTSD that promotes emotional processing of traumatic events through systematic exposure (Foa et al., 2019). Standard PE consists of 10–15 treatment sessions that are 90 min each and delivered weekly over several months. PE includes four primary intervention components: (1) imaginal exposure and processing; (2) in vivo exposure; (3) relaxed breathing; and (4) psychoeducation.

### 2.2. Massed Prolonged Exposure (M-PE)

M-PE included all components of standard PE, but was delivered in a daily format of 15, 90 min sessions over 3 consecutive weeks rather than weekly sessions. Daily sessions have been found to be as effective as once-weekly PE with reduced treatment dropout (Foa et al., 2018). Furthermore, daily treatment may be easier for those, including active-duty service members, who face work-related or other demands that can interfere with treatment when delivered weekly over several months (e.g., military training, deployment, relocation, caregiving responsibilities, and difficulties obtaining release time from work for weekly appointments), or for those who prefer to complete treatment in a shorter period of time.

### 2.3. Intensive Outpatient Program-Prolonged Exposure (IOP-PE)

The IOP-PE also included all standard components of PE and was delivered in a daily format of 15, 90 min sessions over 3 weeks. The IOP-PE was a full day program that also included eight additional treatment components hypothesized to address the needs of military personnel. The IOP-PE components included: (1) a team-based treatment approach for continuity of care and trauma processing with different therapists; (2) clinic-based homework, providing a structured, supportive environment to mitigate avoidance; (3) two daily feedback sessions to promote processing and immediate reinforcement; (4) incorporation of significant others at session two for social support and to minimize accommodation of avoidance; (5) addressing up to three trauma memories during treatment to expand processing; (6) a hierarchical imaginal exposure approach to increase self-efficacy to recount trauma memories; (7) an optional telescopic overview at the final session to broadly reflect on trauma memories; and (8) post-treatment booster sessions to maintain treatment gains. Additional details about the research methods and treatment components for this study can be found in the Methods and Main Outcomes manuscripts (Peterson et al., 2018, 2023).

## 3. Measures

### 3.1. Demographic and Military Characteristics

Information on demographic and military service characteristics was collected at baseline and included items related to demographics (e.g., sex, race, age, and marital status) and military service (e.g., branch of service, military pay grade, military occupation, and number of deployments).

### 3.2. PTSD

PTSD Checklist for DSM-5 (PCL-5; Weathers et al., 2013b). The PCL-5 is a 20-item, self-report assessment of PTSD symptoms. Items are rated using a 5-point Likert scale from 0 (not at all) to 4 (extremely). Total scores range from 0 to 80, with higher scores indicating a higher level of PTSD symptom severity. The PCL-5 has good internal consistency (Cronbach’s α = 0.96), test–retest reliability (*r* = 0.84), and validity (Bovin et al., 2016). The PCL-5 was administered at baseline, weekly during treatment, and at the 1-, 3-, and 6-month follow-ups. In the present sample, Cronbach’s alpha was 0.89 at baseline.

Clinician Administered PTSD Scale for DSM-5 (CAPS-5; Weathers et al., 2013a). The CAPS-5 is a structured clinical interview. Items are rated on a 5-point Likert scale ranging from 0 (absent) to 4 (extreme/incapacitating). The CAPS-5 includes a total score ranging from 0 to 80 with higher scores reflective of greater PTSD severity. The CAPS-5 also assesses DSM–5 criteria for a PTSD diagnosis (i.e., presence of at least one intrusion symptom, one avoidance symptom, two cognition and mood symptoms, and two arousal symptoms for a period of at least one month). The CAPS-5 has excellent psychometric properties and diagnostic efficiency (Weathers et al., 2013a). It was administered at baseline and at the 1-, 3-, and 6-month follow-ups. The baseline internal consistency for the CAPS-5 was 0.76.

### 3.3. Hazardous Drinking

Alcohol Use Disorders Identification Test (AUDIT; Babor et al., 2001). The AUDIT is a 10-item measure developed by researchers at the World Health Organization (WHO) to assess alcohol use and negative consequences associated with drinking. Items are rated on a 5-point Likert scale ranging from 0 to 4. Total scores range from 0 to 40, with higher scores indicating a higher likelihood of problematic drinking and scores of 8 or higher indicating the likely presence of an alcohol use disorder (Babor et al., 2001). The AUDIT has good internal consistency (Cronbach’s α = 0.80), test–retest reliability (*r* = 0.86), and validity (Fleming et al., 1991; Sinclair et al., 1992). The AUDIT was administered at baseline as a screener for problematic drinking. In the present sample, the AUDIT total score internal consistency was 0.85.

Quick Drinking Screen (QDS; Sobell et al., 2003). The QDS is a brief measure that assesses different drinking behaviors per week over an aggregated time period (past 2 weeks in this study). Items evaluate mean number of drinking days, mean number of drinks on drinking days, number of heavy drinking days, and greatest number of drinks in one day. The mean number of total drinks consumed can be calculated by multiplying drinking days by drinks per day. Hazardous (problematic) drinking can be determined based on the presence of a heavy drinking day (4 or more drinks for men, 3 or more for women) and/or a heavy drinking week (14 drinks for men, 7 for women) according to the National Institute on Alcohol Abuse and Alcoholism (NIAAA). The QDS was administered at baseline and at the 1-, 3-, and 6-month post-treatment follow-ups.

## 4. Data Analysis Plan

Prior to addressing the study aims, we calculated descriptives to examine the prevalence and nature of hazardous drinking at baseline. For this study, hazardous drinking (present or absent) at baseline was based on the classifications described above by the WHO (AUDIT ≥ 8) and the NIAAA (heavy drinking on the QDS). If an individual endorsed either of these criteria at baseline, they were classified as meeting criteria for hazardous drinking. We also evaluated group differences across demographic and military characteristics using the chi-square (χ2) test for independence (categorical outcomes) and one-way ANOVA (continuous outcomes), partitioning individuals into 4 groups based on baseline drinking classification and treatment (Table 1). Analyses were intent-to-treat unless the model only included follow-up data. Hypothesis tests were two-tailed with alpha set at 0.05. All analyses were completed using R Version 4.3.3 statistical software.

To test Hypothesis 1 and 2, we used general linear mixed-effects regression models with repeated measures and robust estimation maximum likelihood. Statistical models for the first hypothesis included the effects of treatment (IOP-PE or M-PE), baseline drinking classification (present vs. absent), time (all available visits), and the respective two- and three-way interactions. Baseline PTSD severity was included in models as a covariate. The primary outcome to address Hypothesis 1 was PTSD severity as measured by the PCL-5 and CAPS-5 total scores. The model effect of interest was the three-way interaction of time, treatment, and drinking classification. If the three-way interaction was null, we evaluated the two-way interaction of drinking classification × time and the main effects of time. Pairwise post hoc analyses with a Sidak correction were used to deconstruct significant interactions with an emphasis on group differences in change from baseline to the 1-, 3-, and 6-month follow-ups. We also evaluated PTSD remission on the CAPS-5 using generalized linear models that included the effects of treatment and drinking classification at 1-, 3-, and 6-month follow-up. The effect of interest was the two-way interaction, but lower order effects were examined if the interaction was null.

Hypothesis 2 also employed general linear mixed-effects regression models as described above but only included participants with baseline hazardous drinking. Individuals without hazardous drinking at baseline were not included in outcome analyses because this group was statistically and conceptually different from individuals with hazardous drinking on baseline drinking severity (see Section 5. Results). Comparison of change scores between groups that substantially differ at baseline or when one group demonstrates a floor effect, risks misinterpretation of findings due to treatment based on statistical artifacts (Lord, 1967). Models included the effects of treatment, time, and the two-way interaction. The outcome to address Hypothesis 2 was the QDS and included the following: (a) total drinks per week, (b) number of drinking days, (c) number of drinks per drinking day, (d) number of heavy drinking days (4 ≥ men; 3 ≥ women), and (e) number of drinks per heavy drinking day.

Hypothesis 3 was addressed in two steps. First, we examined group difference in treatment completion rate using a generalized linear model that included the effects of treatment, drinking classification, and the two-way interaction. A treatment completer (binomial) was defined as completion of all 15 sessions or early response (*n* = 33) after at least 12 treatment sessions (≥10-point PCL-5 reduction and participant/provider agreement that early termination was appropriate). The effect of interest was the two-way interaction effect, but lower order effects were examined if the interaction was null. Second, we conducted Kaplan–Meier survival analysis to evaluate group differences in the cumulative proportion of attrition over time. The survival model included the effects of treatment and baseline drinking classification. Time-to-event was defined as the number of sessions completed prior to attrition. A log-rank χ2 test pooled over strata was used to determine if survival distributions differed. If significant effects emerged, χ2 pairwise comparisons were used to further examine the omnibus effect.

## 5. Results

### 5.1. Baseline PTSD and Drinking Severity

Table 2 and Table 3 present detailed results of PTSD and drinking severity at each assessment time point stratified by baseline hazardous drinking classification. The mean PTSD severity for the total sample was 50.77 (*SE* = 1.00) on the PCL-5 and 36.44 (*SE* = 0.63) on the CAPS-5 at the baseline assessment. There was not a significant difference in baseline PTSD severity on the PCL-5 or CAPS-5 among individuals with and without hazardous drinking (both *p*’s > 0.05). 

**Table 2:** Estimated Marginal Means of PTSD by Time, Baseline Drinking Classification, and Treatment Format ^a^.

		Hazardous Drinking Absent				Hazardous Drinking Present			
		IOP-PE		M-PE		IOP-PE		M-PE	
Outcome	Time	*M*	*SE*	*M*	*SE*	*M*	*SE*	*M*	*SE*
PCL-5	Baseline	51.28	1.51	50.70	1.43	50.16	2.24	50.96	2.56
	1MFU	26.22	1.95	28.85	1.71	34.70	2.63	35.96	3.13
	3MFU	25.41	1.97	28.41	1.79	32.37	2.86	41.74	3.39
	6MFU	28.88	1.96	30.95	1.92	32.86	2.80	38.70	3.43
CAPS-5	Baseline	36.60	0.95	36.48	0.90	36.26	1.41	36.44	1.61
	1MFU	20.95	1.28	21.65	1.12	26.58	1.72	26.10	2.06
	3MFU	18.72	1.32	22.37	1.16	24.23	1.84	26.30	2.15
	6MFU	21.81	1.33	25.42	1.31	22.63	1.88	28.20	2.34

Note. IOP-PE = Intensive Outpatient Program Prolonged Exposure, M-PE = Massed Prolonged Exposure, PCL-5 = PTSD Checklist for DSM-5, CAPS-5 = Clinician Administered PTSD Scale for DSM-5, MFU = month follow-up. ^a^ Statistical models included baseline PTSD severity. There were not statistically significant differences between groups at baseline.

Overall, 27.4% (*n* = 64) of the sample endorsed baseline hazardous drinking. Among participants with hazardous drinking, 43.8% (*n* = 28) were randomized to M-PE and 56.3% (*n* = 36) to IOP-PE. Individuals with hazardous drinking had an AUDIT total score of 10.29 (*SE* = 0.86) at baseline. During the two weeks prior to baseline, individuals with hazardous drinking in both treatment groups on average consumed 12.89 (*SE* = 1.61) standard drinks per week, drank 3.04 (*SE* = 0.32) days per week, and had 4.56 (*SE* = 0.39) drinks on drinking days. Individuals with hazardous drinking also endorsed 1.39 (*SE* = 0.28) heavy drinking days per week and consumed 5.81 (SE = 0.50) drinks on heavy drinking days. Regarding participants without hazardous drinking (72.6%, *n* = 170), 52.4% (*n* = 89) were randomized to M-PE and 47.6% (*n* = 81) to IOP-PE. Participants without hazardous drinking at baseline consumed 1.57 (*SE* = 0.15) standard drinks per week and had an AUDIT total score of 2.11 (*SE* = 0.14). Individuals without hazardous drinking had significantly lower baseline drinking severity on the AUDIT, *t*(230) = −14.26, *p* < 0.001, and QDS, *t*(230) = 12.01, *p* < 0.001, compared to individuals with hazardous drinking. 

**Table 3:** Estimated Marginal Means of Drinking Patterns by Time and Treatment Format Among Individuals with Baseline Hazardous Drinking.

		Hazardous Drinking Present			
		IOP-PE		M-PE	
QDS	Time	*M*	*SE*	*M*	*SE*
Total Drinks in Past Week	Baseline	12.69	2.13	13.09	2.42
	1MFU	7.97	2.54	10.66	3.03
	3MFU	4.04	2.62	12.10	3.05
	6MFU	6.70	2.85	8.35	3.47
Past Week Drinking Days	Baseline	2.83	0.38	3.24	0.43
	1MFU	2.54	0.44	2.79	0.51
	3MFU	1.84	0.44	2.45	0.51
	6MFU	2.29	0.48	1.88	0.57
Past Week Drinks per Drinking Day	Baseline	4.74	0.51	4.74	0.51
	1MFU	2.37	0.63	2.37	0.63
	3MFU	1.62	0.70	1.62	0.70
	6MFU	2.61	0.74	2.61	0.74
Past Week Heavy Drinking Days	Baseline	1.48	0.37	1.29	0.42
	1MFU	1.25	0.45	0.84	0.54
	3MFU	0.39	0.50	1.63	0.58
	6MFU	1.03	0.52	0.59	0.64
Past Week Drinks per Heavy Drinking Day	Baseline	5.90	0.65	5.73	0.74
	1MFU	3.05	0.79	3.31	0.95
	3MFU	1.83	0.83	3.34	0.97
	6MFU	3.34	0.90	2.34	1.11

Note. IOP-PE = Intensive Outpatient Program Prolonged Exposure, M-PE = Massed Prolonged Exposure, QDS = Quick Drinking Screen, MFU = month follow-up.

### 5.2. PTSD Outcomes

Table 4 presents model-estimated PTSD and drinking severity reductions since baseline stratified by baseline hazardous drinking classification. No significant three-way interaction (treatment × hazardous drinking classification × time) was found on the PCL-5, *F* (6, 888) = 0.61, *p* = 0.721, indicating that PTSD symptom reductions did not vary as a function of both treatment and drinking classification. There was, however, a significant two-way interaction of drinking classification and time on the PCL-5, *F* (6, 888) = 3.37, *p* = 0.003). That is, individuals without hazardous drinking demonstrated greater PTSD symptom reductions compared to those with hazardous drinking at the 1-month (*M_diff_* = 8.23, *p* = 0.001), 3-month (*M_diff_* = 10.57, *p* < 0.001), and 6-month *M_diff_* = 6.29, *p* = 0.011) follow-ups.

There was also no significant three-way interaction (treatment × hazardous drinking × time) on the CAPS-5 total score, *F* (3, 347) = 0.30, *p* = 0.823. However, there was a two-way interaction effect (drinking classification × time), *F* (3, 347) = 4.17, *p* = 0.006. Participants without hazardous drinking had greater PTSD symptom reductions compared to the hazardous drinking group on the CAPS-5 at the 1-month (*M_diff_* = 5.23, *p* = 0.004) and 3-month (*M_diff_* = 4.92, *p* = 0.005) follow-ups, but this difference dissipated at the final follow-up (*M_diff_* = 2.00, *p* = 0.306). Importantly, although individuals without hazardous drinking had greater PTSD symptom reductions, participants experienced statistically significant improvements following treatment regardless of PE treatment format or hazardous drinking classification (main effect of time) on the PCL-5, *F* (6, 888) = 113.81, *p* < 0.001, and CAPS-5, *F* (3, 347) = 181.41, *p* < 0.001. There was also a notable PTSD remission rate among individuals who participated in follow-up assessments at 1-month (55.6%), 3-month (57.6%), and 6-month (51.9%) follow-up. Individuals without hazardous drinking demonstrated greater PTSD remission rates than those with hazardous drinking at the 1-month (61.5% vs. 41.0%; *χ*2 (1, *N* = 135) = 4.82, *p* = 0.028) and 6-month (58.7% vs. 35.5%; *χ*2 (1, *N* = 106) = 5.06, *p* = 0.024) follow-up.

**Table 4:** PTSD and Drinking Severity Reductions from Baseline to Follow-Up by Baseline Hazardous Drinking Classification.

	**Hazardous Drinking Absent**		**Hazardous Drinking Present**		**Haz x Time**		**Group Differences in Change**			
**PTSD Severity**	***M***	***SE***	***M***	***SE***	***F***	***p***	***M_diff_***	***t***	***p***	***d***
PCL-5					3.37	0.003				
Baseline to 1MFU	−23.46	1.37	−15.23	2.17			8.23	3.21	0.001	0.42
Baseline to 3MFU	−24.07	1.34	−13.51	2.24			10.57	4.05	<0.001	0.53
Baseline to 6MFU	−21.07	1.31	−14.78	2.10			6.29	2.54	0.011	0.33
CAPS-5					4.17	0.018				
Baseline to 1MFU	−15.24	0.95	−10.01	1.51			5.23	2.93	0.004	0.38
Baseline to 3MFU	−15.99	0.93	−11.08	1.49			4.92	2.81	0.005	0.37
Baseline to 6MFU	−12.93	1.03	−10.93	1.65			2.00	1.03	0.306	0.14
	**Hazardous Drinking Absent**		**Hazardous Drinking Present**		**Arm x Time**		**Simple Main Effect of Time**			
**Drinking Patterns**	***M***	***SE***	***M***	***SE***	***F***	***p***		***t***	***p***	***d***
**Past Week Total Drinks**					1.42	0.237				
Baseline to 1MFU	---	---	−3.57	2.38				−1.70	0.092	−0.42
Baseline to 3MFU	---	---	−4.82	1.91				−2.52	0.013	−0.57
Baseline to 6MFU	---	---	−5.36	2.36				−2.27	0.025	−0.63
**Past Week Drinking Days**					0.63	0.597				
Baseline to 1MFU	---	---	−0.37	0.32				−1.17	0.244	−0.29
Baseline to 3MFU	---	---	−0.89	0.28				−3.17	0.002	−0.72
Baseline to 6MFU	---	---	−0.95	0.35				−2.69	0.008	−0.77
**Past Week Drinks per Drinking Day**					0.66	0.582				
Baseline to 1MFU	---	---	−2.21	0.65				−3.41	<0.001	−0.77
Baseline to 3MFU	---	---	−2.24	0.64				−3.77	<0.001	−0.84
Baseline to 6MFU	---	---	−2.53	0.72				−3.49	<0.001	−0.87
**Past Week Heavy Drinking Days**					1.51	0.218				
Baseline to 1MFU	---	---	−0.34	0.38				−0.90	0.369	−0.18
Baseline to 3MFU	---	---	−0.38	0.42				−0.91	0.369	−0.20
Baseline to 6MFU	---	---	−0.58	0.44				−1.32	0.189	−0.31
**Past Week Drinks on Heavy Drinking Days**					0.83	0.481				
Baseline to 1MFU	---	---	−2.64	0.70				−3.77	<0.001	−0.91
Baseline to 3MFU	---	---	−3.22	0.66				−4.90	<0.001	−1.11
Baseline to 6MFU	---	---	−2.97	0.79				−3.77	<0.001	−1.03

Notes. IOP-PE = Intensive Outpatient Program Prolonged Exposure, M-PE = Massed Prolonged Exposure, Haz = Hazardous Drinking Classification, PCL-5 = PTSD Checklist for DSM-5, CAPS-5 = Clinician Administered PTSD Scale for DSM-5, MFU = month follow-up, *d* = Cohen’s *d*, *M_diff_* = between group difference in change from BL to FU (a positive *M_diff_* reflects greater severity reductions in the Hazardous Drinking Absent Classification compared to the Hazardous Drinking Positive Classification).

### 5.3. Drinking Outcomes

Among individuals with baseline hazardous drinking, there were significant reductions following treatment in the mean total number of drinks per week, *F* (3, 95) = 3.73, *p* = 0.014, number of drinking days per week, *F* (3, 95) = 5.42, *p* = 0.002, drinks consumed on drinking days per week, *F* (3, 95) = 7.64, *p* < 0.001, and drinks consumed on heavy drinking days per week, *F* (3, 95) = 10.88, *p* < 0.001. As seen in Table 4, individuals had 4.82 (*SE* = 1.91) fewer total drinks per week at the 3-month follow-up and 5.36 (*SE* = 2.36) fewer at the 6-month follow-up (both *p*’s < 0.05). Additionally, participants reported 0.89 (*SE* = 0.28) fewer drinking days per week at the 3-month follow-up and 0.95 (*SE* = 0.35) fewer at the 6-month follow-up (both *p*’s < 0.01). Individuals with hazardous drinking also consumed approximately two fewer drinks per drinking day and three fewer drinks on heavy drinking days at the 1-, 3-, and 6-month post-treatment follow-ups compared to the baseline assessment (all *p*’s < 0.01). However, following treatment, there was not a significant reduction in the number of heavy drinking days per week, *F*(3, 95) = 0.75, *p* = 0.527. As seen in Table 4, there were no significant two-way interactions (treatment format × time) found on the QDS, suggesting drinking outcomes did not vary by PE treatment formats (all *p*’s > 0.05).

### 5.4. Treatment Engagement Outcomes

**Figure 1:**
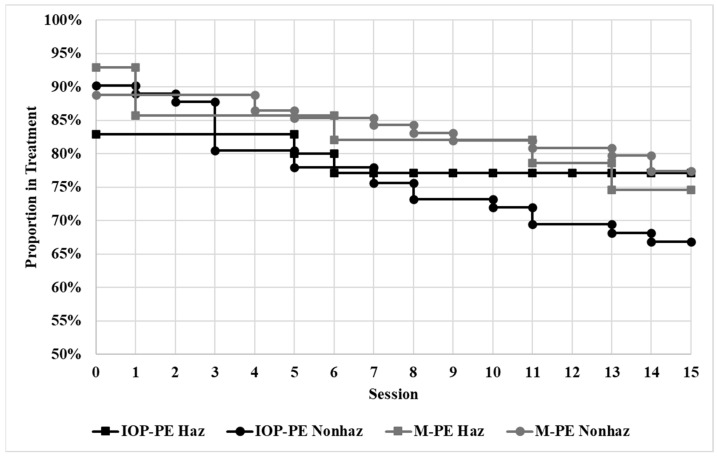
Survival Analysis of Treatment Engagement by Drinking Classification and Treatment Format. Note. IOP-PE = Intensive Outpatient Program Prolonged Exposure, M-PE = Massed Prolonged Exposure, Haz = Hazardous Drinking Present, Nonhaz = Hazardous Drinking Absent.

Overall, 74.4% of the sample completed treatment. The average number of sessions attended in the sample was 11.88 (*SE* = 0.35) and half of the participants who dropped out of treatment did so prior to beginning treatment (49.1%; Figure 1). There were no significant two-way interactions (drinking classification × treatment) on treatment completion rate, *χ*2 (1, *N* = 234) = 1.15, *p* = 0.284, or time to dropout, *χ*2 (1, *N* = 234) = 0.31, *p* = 0.580. There were also no significant main effects of drinking classification on treatment completion, *χ*2 (1, *N* = 234) = 0.46, *p* = 0.500, or time to dropout, *χ*2 (1, *N* = 234) = 0.24, *p* = 0.628.

## 6. Discussion

This study was a secondary analysis that examined the impact of co-occurring hazardous drinking on PTSD symptom improvement and treatment engagement. Data used were from a parent randomized controlled trial that tested two formats of PE for PTSD (IOP-PE and M-PE). We also evaluated drinking reductions among those with hazardous drinking and whether outcomes varied as a function of PE treatment format. Consistent with prior research, a considerable proportion (approximately 1 in 4) of participants endorsed hazardous drinking at baseline (Jakupcak et al., 2010). Regardless of drinking classification or PE treatment format, participants experienced significant reductions in PTSD symptoms following treatment. However, individuals without hazardous drinking had better PTSD outcomes compared to those with hazardous drinking, indicating that alcohol use impacted PTSD treatment response. Contrary to our hypothesis, participants with hazardous drinking in M-PE did not have less PTSD reductions compared to those in IOP-PE, suggesting that both PE treatment formats were effective in addressing PTSD. These findings support the known benefits of trauma-focused psychotherapies for PTSD and underscore that PE can safely and effectively reduce PTSD symptoms regardless of drinking behaviors. The attenuated PTSD treatment benefits observed in the hazardous drinking group relative to the group without hazardous drinking were moderate in magnitude. This highlights how alcohol use may serve as an avoidance mechanism that impairs emotional processing (e.g., reduced arousal) and inhibitory learning (e.g., difficulties in the acquisition, retention, and application of therapeutic skills) during PE (Craske et al., 2014; Foa et al., 2019). The presence of drinking also increases risk for additional mental health and physical health problems that can further complicate treatment and impact overall well-being. Nonetheless, the hazardous drinking group still exhibited significant improvements in PTSD symptoms, indicating that PE can yield meaningful benefits despite the presence of hazardous drinking.

Individuals with hazardous drinking in this study also demonstrated significant reductions in alcohol use across all drinking outcomes following treatment, with the exception of the number of heavy drinking days per week. Contrary to our hypothesis, there were no significant effects of treatment across drinking outcomes, indicating that both PE formats produced comparable drinking reductions. Improvements in alcohol use from PE alone are encouraging, as even modest decreases in drinking can contribute to improved functioning, well-being, and PTSD treatment outcomes and engagement consistent with harm-reduction research (Marlatt & Witkiewitz, 2002). Results also demonstrate that addressing PTSD is associated with decreased drinking even though reducing alcohol use is not a core intervention component addressed in PE. The lack of significant changes in number of heavy drinking days in this study may be attributable to drinking patterns in the sample. That is, individuals with hazardous drinking in this study generally demonstrated a pattern of heavy, binge drinking on one or two days per week. However, it is important to note that there were significant reductions in the number of drinks consumed on drinking days and number of drinks consumed on heavy drinking days following treatment. Therefore, although the frequency of heavy drinking days did not significantly improve, participants did have reductions in heavy, binge drinking patterns as evidenced by fewer drinks consumed on drinking days following treatment. Alternatively, the absence of improvement in the number of heavy drinking days per week may have been constrained by floor effects that limited the potential for improvement or indicate only partial improvement, highlighting the need for additional interventions that target co-occurring hazardous drinking.

Although differences in PTSD treatment benefits were observed between those with and without hazardous drinking, drinking classification was not associated with treatment engagement in this study. This finding increases confidence that individuals with PTSD and hazardous drinking can tolerate trauma-focused psychotherapies, such as PE. Overall, attrition was quite low regardless of drinking classification or treatment format. The low attrition rates and absence of group differences may be attributable to the known benefits of daily, massed treatment formats (Foa et al., 2018; Held et al., 2021). Treatment engagement outcomes in the present study provide additional support for compressed treatment formats and indicate that the benefits of massed treatments may generalize to individuals with PTSD and hazardous drinking.

These findings have several important clinical implications. First, clinicians should routinely assess alcohol use in individuals with PTSD, as the co-occurrence of hazardous drinking is prevalent and may impact treatment outcomes. Baseline assessment of alcohol use can facilitate targeted psychoeducation regarding the relationship between PTSD and alcohol. It also enables monitoring of changes in drinking during treatment and the integration of alcohol-related factors into PE intervention components (e.g., drinking in temporal or contextual proximity to exposure or trauma cues). Second, the results demonstrate that individuals with PTSD and hazardous drinking can benefit from PE alone when delivered in compressed formats. That said, differences in PTSD response compared to individuals without hazardous drinking, and lack of improvement across select drinking outcomes, align with the self-medication hypothesis where hazardous drinking operates as a maladaptive avoidance behavior that attenuates emotional and cognitive processing during PE. This trend may also highlight the utility of an integrated treatment, such as COPE. However, because this study did not include an integrated treatment, definitive conclusions about the relative advantages or disadvantages of PE alone are preliminary. Another consideration is that PE alone may serve as a harm-reduction approach for individuals with PTSD and alcohol misuse who are not ready or willing to engage in an integrated treatment or to address their drinking. Finally, several CPT studies have not found significant differences in PTSD treatment response among individuals with and without hazardous drinking (Dondanville et al., 2019; Held et al., 2021; LoSavio et al., 2023; Straud et al., 2021). It is unclear why differential effects among individuals with and without hazardous drinking were observed in this study and not in prior CPT research given that both have strong efficacy in treating PTSD. A common limitation across exploratory, secondary studies is unequal groups and small cell sizes among hazardous drinking groups. This trend was observed in the present study and the prior CPT studies, and it could be an explanation for divergent results.

These findings also have implications for future research directions. First, future research should consider a randomized controlled trial to compare PE alone to COPE or PE with AUD medications among individuals with co-occurring PTSD and hazardous drinking. This would allow for examination of the incremental effects of integrated substance use interventions on PTSD and substance use outcomes. Second, future research should evaluate if study findings generalize more broadly to patterns of problematic drinking. Although we used a recognized definition, hazardous drinking can be operationalized using a number of approaches and alternative classifications may have yielded different results. Third, additional research is needed to identify factors that moderate or mitigate the disparity in PTSD treatment outcomes between individuals with and without hazardous drinking engaged in trauma-focused psychotherapy. Finally, future research should examine how individual-level predictors and patient preferences can guide optimal treatment selection, enhance personalized care, and maximize outcomes.

This study had several limitations and findings should be interpreted within this context. First, the sample included individuals who endorsed hazardous drinking, but this is not necessarily reflective of alcohol use disorder and results may not generalize to more severe drinking populations. Hazardous drinking classification was also based on self-report and a clinician-administered interview would have been a preferred approach. Another limitation was that hazardous drinking classification groups in this study were relatively small, and cell sizes were unequal across the groups of interest. These factors reduce statistical power and limit the ability to detect group differences, interactions, and moderator effects. Another limitation was the absence of a comparison group, treatment as usual or integrated treatment (COPE), which would have strengthened conclusions regarding treatment effects. A final limitation is that the sample was recruited from Texas and may not generalize to the broader U.S. military, veteran, or civilian populations.

Study findings are important given high rates of co-occurring PTSD and hazardous drinking in U.S. military personnel and veterans, as well as research demonstrating that the co-occurrence of PTSD and hazardous drinking is associated with poorer treatment outcomes and decreased treatment engagement. Our results suggest that individuals with hazardous drinking experience less PTSD symptom reductions than those without hazardous drinking, although they still achieve meaningful PTSD improvement and reductions in some drinking outcomes following PE. This underscores the robustness of PE as a treatment for co-occurring PTSD and alcohol misuse when delivered in either M-PE or IOP-PE format, at least among a mild to moderate severity, binge drinking sample. Importantly, this study provides additional evidence that individuals with hazardous drinking can tolerate PE. Findings are also consistent with research showing that compressed treatment formats result in more individuals completing treatment and potentially generalizability of this finding to those with PTSD and problematic drinking. Overall, this study suggests that PE is an appropriate treatment for individuals with PTSD and hazardous drinking and that PE delivered in a compressed format is a viable option when available and possible for the patient.

## Figures and Tables

**Table 1:** Demographics and Military Characteristics ^a^.

			Hazardous Drinking Absent				Hazardous Drinking Present					
Characteristic	Total (*N* = 234)		IOP-PE (*n* = 81)		M-PE (*n* = 89)		IOP-PE (*n* = 36)		M-PE (*n* = 28)			
	*M*	*SE*	*M*	*SE*	*M*	*SE*	*M*	*SE*	*M*	*SE*	*F*	*p*
**Age**	39.20	0.84	38.99	0.85	38.99	0.84	39.86	1.10	39.18	1.732	0.11	0.954
**Months in military**	181.88	5.42	185.06	8.82	182.08	9.19	182.08	12.52	171.79	17.42	0.18	0.911
	***N***	***%***	***N***	***%***	***N***	***%***	***N***	***%***	***N***	***%***	**χ2**	***p***
**Men**	182	77.8	62	76.5	68	76.4	31	86.1	21	75.0	1.54	0.673
**Married**	151	64.5	56	69.1	57	64.0	24	66.7	14	50.0	3.50	0.320
**Ethnicity**											5.99	0.741
Caucasian/Non-Hispanic	102	43.6	29	35.8	41	46.6	16	44.4	16	59.3		
African American	61	26.1	23	28.4	23	26.1	10	27.8	5	18.5		
Hispanic	58	24.7	22	27.2	23	26.1	8	22.2	5	18.5		
Other	7	3.0	4	4.9	1	1.1	1	2.8	1	3.7		
**Education**											4.14	0.658
High school or less	14	5.95	6	7.4	5	5.6	3	8.3	0	0.0		
Some college/associate degree	144	61.5	53	65.4	53	59.6	21	58.3	17	60.7		
College/graduate degree	76	32.5	22	27.1	31	34.8	12	33.3	11	39.3		
**Active ^b^**	152	65.0	55	67.9	56	62.9	22	61.1	19	67.9	1.04	0.791
**Army**	191	81.6	69	85.1	75	84.3	23	63.9	24	85.7	6.98	0.072
**Enlisted rank**	200	85.5	70	86.4	76	85.3	31	86.1	23	82.1	3.52	0.741
**Military Occupation**											6.36	0.384
Combat Arms	81	34.6	32	39.5	23	25.8	15	41.7	11	39.3		
Combat Support	63	26.9	22	27.2	25	28.1	10	27.8	6	21.4		
Combat Service Support	89	38.0	26	32.1	41	46.1	11	30.6	11	39.3		
**Number of deployments**											6.36	0.384
1	69	29.5	20	24.7	29	32.6	10	25.0	11	39.3		
2	70	29.9	27	33.3	29	32.6	7	19.4	7	25.0		
3	48	20.5	17	21.0	16	18.0	9	25.0	6	21.4		
4+	46	19.7	17	21.0	15	16.9	10	27.8	4	14.3		

Note. IOP-PE = Intensive Outpatient Program Prolonged Exposure, M-PE = Massed Prolonged Exposure. Cell counts vary based on available data across variables. ^a^ *F* and χ2 analyses partitioned individuals into 4 groups based on baseline drinking classification and treatment format to test for differences across demographic and military characteristics. ^b^ Seven participants in this category were serving in the U.S. Army Reserve or National Guard.

## Data Availability

The data presented in this study are available upon request through the STRONG STAR Repository, maintained at The University of Texas Health Science Center at San Antonio (San Antonio, TX, USA). Requests related to the Repository should be submitted to repository@strongstar.org.
